# Efficacy of levocetirizine in isolated rat tracheal smooth muscle

**DOI:** 10.7150/ijms.86769

**Published:** 2023-10-02

**Authors:** Ying-Liang Chou, Yueh-Ting Wang, Li-Hsiang Cheng, Shao-Cheng Liu, Hsing-Won Wang

**Affiliations:** 1 Department of Otolaryngology-Head and Neck Surgery, Taichung Armed Forces General Hospital, Taichung, Taiwan, Republic of China.; 2 Department of Otolaryngology-Head and Neck Surgery, Tri-Service General Hospital, National Defense Medical Center, Taipei, Taiwan, Republic of China.; 3 Department of Radical Imaging and Radiological Sciences, Central Taiwan University of Science and Technology, Taichung, Taiwan, Republic of China.; 4 Bioinformatics Program, Boston University, Boston, MA, USA.; 5 The Graduate Institute of Clinical Medicine and Department of Otolaryngology, College of Medicine, Taipei Medical University-Shuang Ho Hospital, Taipei, Taiwan, Republic of China.

**Keywords:** levocetirizine, menthol, trachea, smooth muscle, cold receptor, in vitro study

## Abstract

Histamine receptor-1 (H_1_) antagonists like levocetirizine are frequently used nowadays to treat rhinitis patients who experience rhinorrhea and sneezing. The trachea may be affected by the H_1_ antagonist when it is used to treat nasal symptoms, either orally or through inhalation. The purpose of this study was to ascertain in vitro effects of levocetirizine on isolated tracheal smooth muscle. As a parasympathetic mimetic, methacholine (10^-6^ M) causes contractions in tracheal smooth muscle, which is how we tested effectiveness of levocetirizine on isolated rat tracheal smooth muscle. We also tested the drug's impact on electrically induced tracheal smooth muscle contractions. The impact of menthol (either before or after) on the contraction brought on by 10^-6^ M methacholine was also investigated. According to the results, the addition of levocetirizine at concentrations of 10^-5^ M or more caused a slight relaxation in response to methacholine's 10^-6^ M contraction. Levocetirizine could prevent spike contraction brought on by electrical field stimulation (EFS). As the concentration rose, it alone had a neglect effect on the trachea's basal tension. Before menthol was applied, levocetirizine might have also inhibited the function of the cold receptor. According to this study, levocetirizine might potentially impede the parasympathetic function of the trachea. If levocetirizine was used prior to menthol addition, it also reduced the function of cold receptors.

## Introduction

Smooth muscle contractility has been examined in vitro using vascular and nasal mucosal strips 1, 2. This study tested the effects of medications that cause tracheal constriction or relaxation using rat tracheas and a straightforward in vitro approach. Antihistamine usage in asthma has long been debatable. The early first-generation antihistamines were ineffective at the levels advised for allergic rhinitis, while larger doses resulted in terrible side effects, making them useless in the treatment of asthma. Their usage, intravenously or by aerosol, was constrained by these adverse effects 3, 4. It has been suggested that second- or third-generation antihistamines may aid in the management of asthma 5, 6.

Since histamine plays a significant and varied role in the pathogenesis of allergic illness, treatment intervention is often geared toward limiting histamine's effects. Considered a second-generation antihistamine, levocetirizine is widely prescribed for a variety of allergy conditions. Investigating the function of levocetirizine in asthma makes sense given that it is one of these allergic illnesses. A straightforward in vitro model was applied to rat tracheas considering our understanding of the effects of levocetirizine on tracheal constriction or relaxation.

Cold-induced airway hyperresponsiveness is common in people with bronchial asthma and is an issue for those who live in cold climates. The main cold temperature sensor in humans is the transient receptor potential melastatin 8 (TRPM8) channels, which may mediate cold response in asthmatics with cold-induced airway hyperresponsiveness 7, 8. The interaction between antihistamines and cold receptors has not received much attention. Given that both antihistamines and cold receptors may influence the muscle's ability to contract, it seems sense to investigate how they interact with isolated tracheal smooth muscle.

The procedure was developed from a method that had already been described 9 and involved suspending 5-mm strips of rat trachea in a tissue bath containing 30 ml of Krebs solution 10,11. The purpose of this study was to ascertain in vitro effects of levocetirizine on isolated tracheal smooth muscle. Investigations were also conducted on the impact of menthol (either before or after) on contractions brought on by 10^-6^ M methacholine.

## Materials and methods

**Figure 1:**
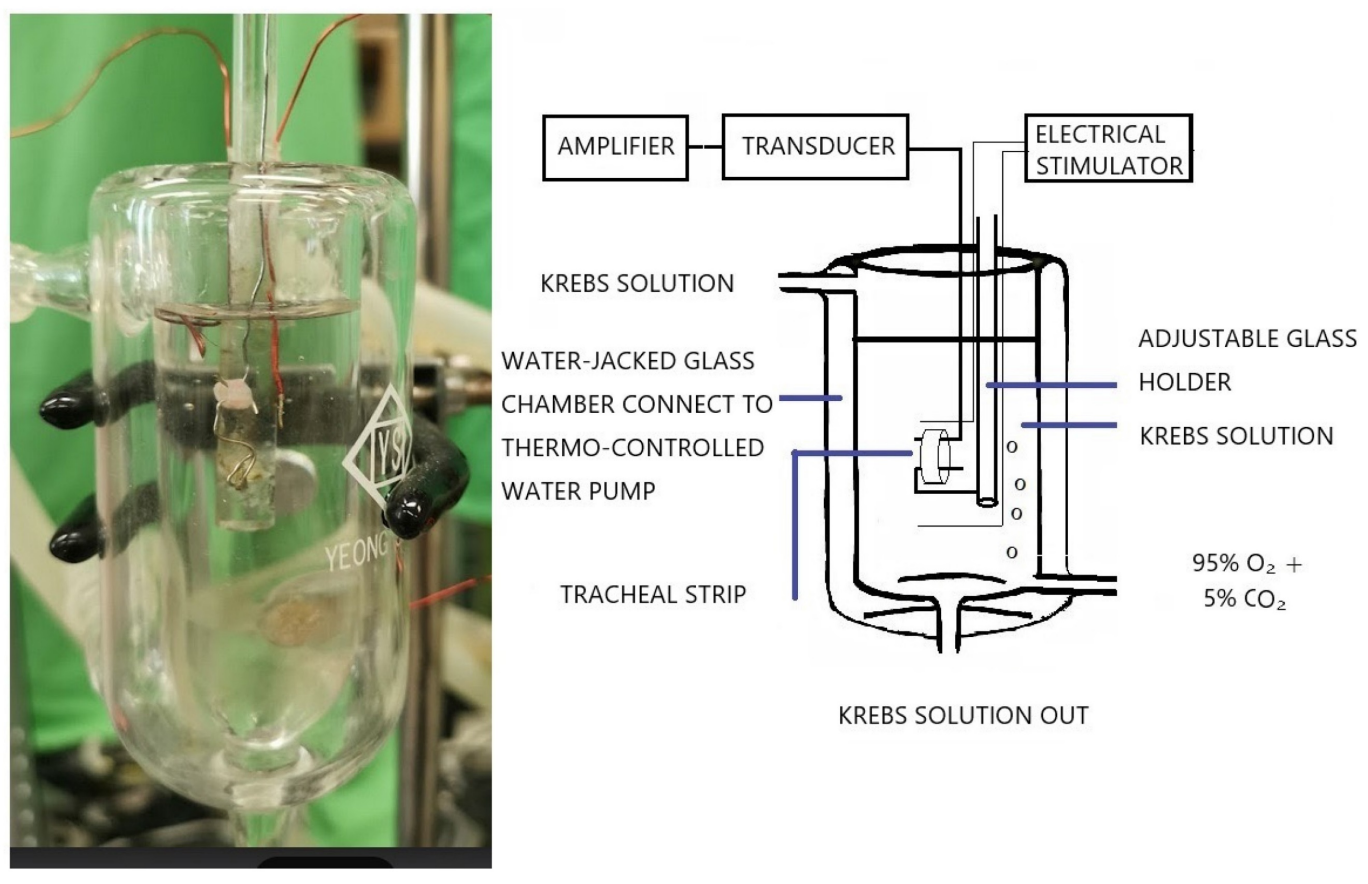
The tension in isolated rat tracheal smooth muscle was measured, as shown in the schematic diagram and actual photo.

The purest chemicals available were used. We were able to get pure levocetirizine powder thanks to UCB Pharmaceuticals (Taiwan) Ltd. The source of all further chemical reagents was from Sigma in St. Louis, Missouri, USA. Methacholine was put to the test as a tracheal contraction aid. The ARRIVE (Animal Research: Reporting of In Vivo Experiments) protocols were followed during the animal experiment. Using CO_2_ gas asphyxiation, 30 healthy male Sprague-Dawley rats were humanely killed, and 2-3 pieces of each rat's trachea (each measuring about 5 mm in length) were extracted. An animal experiment review board (LAC-2016-0242) gave its clearance to this investigation. According to earlier reports, the tracheal specimen was placed using two steel hooks and immersed in a 30 ml muscle bath at 37°C (Fig. 1) 9. Thirty ml of Krebs solution, which contained (in mmol/L) NaCl, 118; KCl, 4.7; CaCl_2_, 2.5; MgSO_4_7H_2_O, 1.2; KH_2_PO_4_, 1.2; NaHCO_3_, 25.0; and glucose, 10.0, was added to the bath. A steel hook and a 3-0 silk ligature were used to secure the upper side of the tracheal strip to a Grass FT-03 force displacement transducer (AstroMed, West Warwick, RI, USA). The strip's other side was fastened to a steel hook that was joined to the bathtub. With the help of Chart V4.2 software (PowerLab, ADInstruments, Colorado Springs, CO, USA), changes in tension were continually recorded after applying a passive stress of 0.3 g to the strips. Preliminary tests showed that the tracheal strip immersed in the bath solution used for subsequent experiments did not contract when basal tension was applied. Before drug assays were conducted, isolated tracheas were equilibrated in the bath solution for 15-30 min, during which continuous aeration with a mixture of 95% O_2_ and 5% CO_2_ was applied. Stepwise increases in the amounts of drugs used were employed to study contraction or relaxation responses of tracheal strips. All drugs were administered by adding a defined volume of stock solution to the tissue bath solution. In each experiment, one untreated strip served as a control.

Two wire electrodes, positioned parallel to the trachea strip and coupled to a direct-current stimulator (Grass S44, Quincy, MA, USA), were used to apply electrical field stimulation (EFS) to the trachea strip at a frequency of 5 Hz, a pulse width of 5 ms, and a voltage of 50 V, with trains of stimulation lasting 5 seconds. Each stimulation period had a 2-minute break in between to give the body time to recover from the response. The trachea was continuously stimulated at a temperature of 37°C.

The effects of levocetirizine on tracheal smooth muscle resting tension, contractions brought on by 10^-6^ M methacholine, and electrically produced tracheal smooth muscle contractions were all evaluated. The impact of menthol (either before or after) on the contraction brought on by 10^-6^ M methacholine was also examined.

The amount of each medication in the 30 ml bath solution was used to express the drug concentrations. Standard deviations (SDs) and mean values were used to present the data. The Student's t-test was used to compare variations in mean values. At P < 0.05, differences were thought to be significant.

## Results

**Figure 2:**
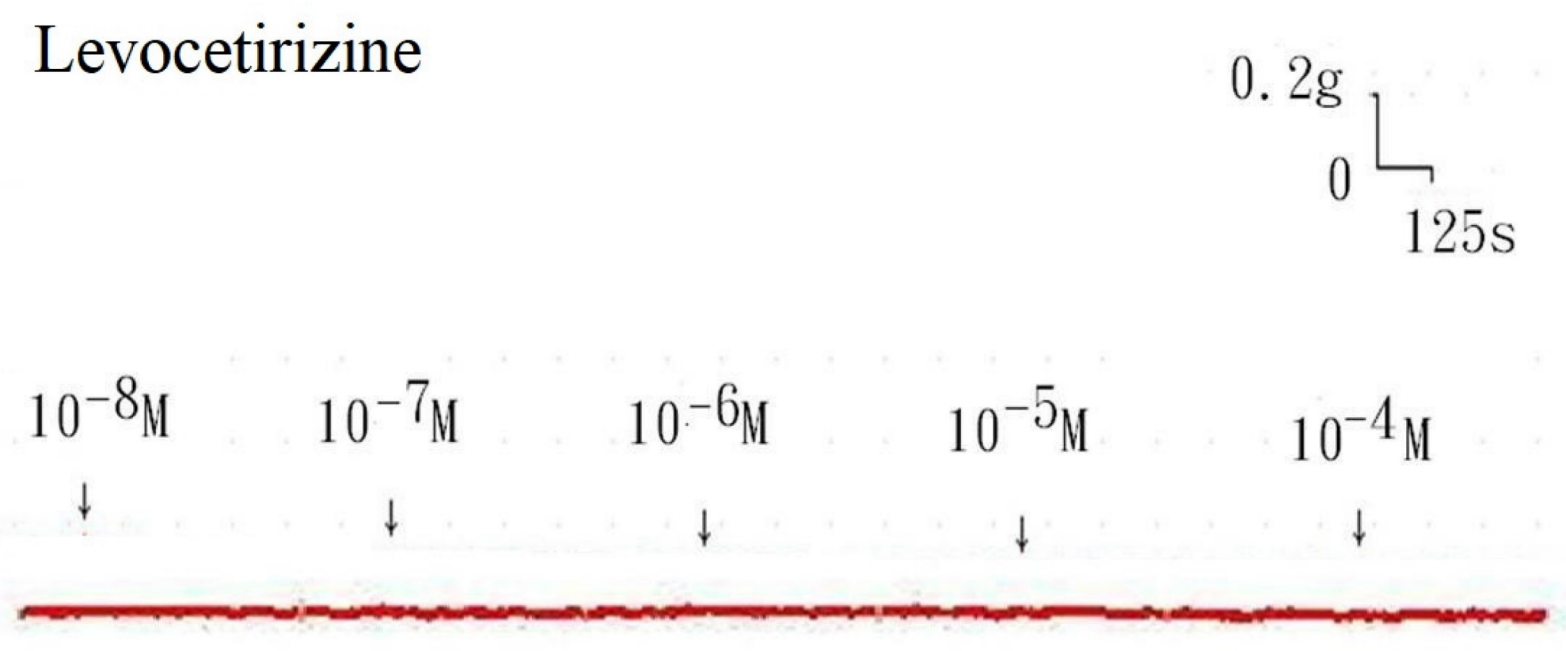
Tension changes in the rat trachea after the application of various levocetirizine concentrations. Basal tension was 0.3 g.

**Figure 3:**
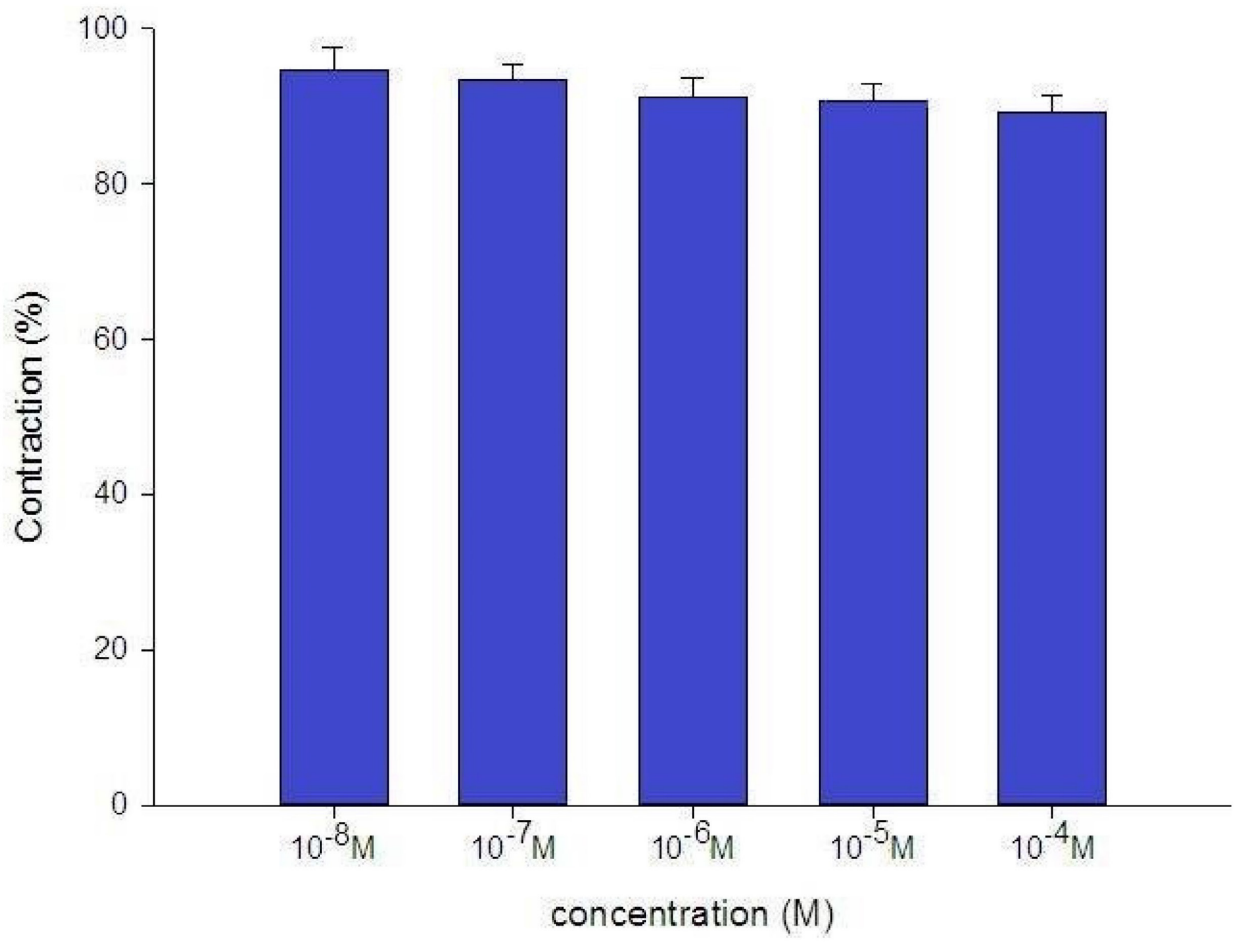
Effects of levocetirizine on 10^-6^ M methacholine-induced contraction (contraction area was calculated at 100% with no addition of levocetirizine) of rat tracheas. The difference of tension between control values and 10^-5^ M or 10^-4^ M levocetirizine was statistically significant (P<0.05). Results were mean + SD (n = 6).

The tension placed on the transducer was used to estimate the degree of tracheal strip relaxation or contraction. Methacholine can easily cause tracheal contraction, and the tissue remains contracted until the medication is removed from the tissue. When administered following the addition of a constricting agent such as 10^-6^ M methacholine, levocetirizine produced a slight relaxation of the trachea but had no effect on the basal tension (Fig. 2). The trachea's smooth muscle was mildly relaxed by higher levocetirizine concentrations (Figs. 3). The tensions were 90.6±4.9% and 89.2±5.2%, of control values respectively, at 10^-5^ M and 10^-4^ M levocetirizine (Fig. 3). There was a statistically significant difference in tension between control values and 10^-5^ M or 10^-4^ M levocetirizine.

**Figure 4:**
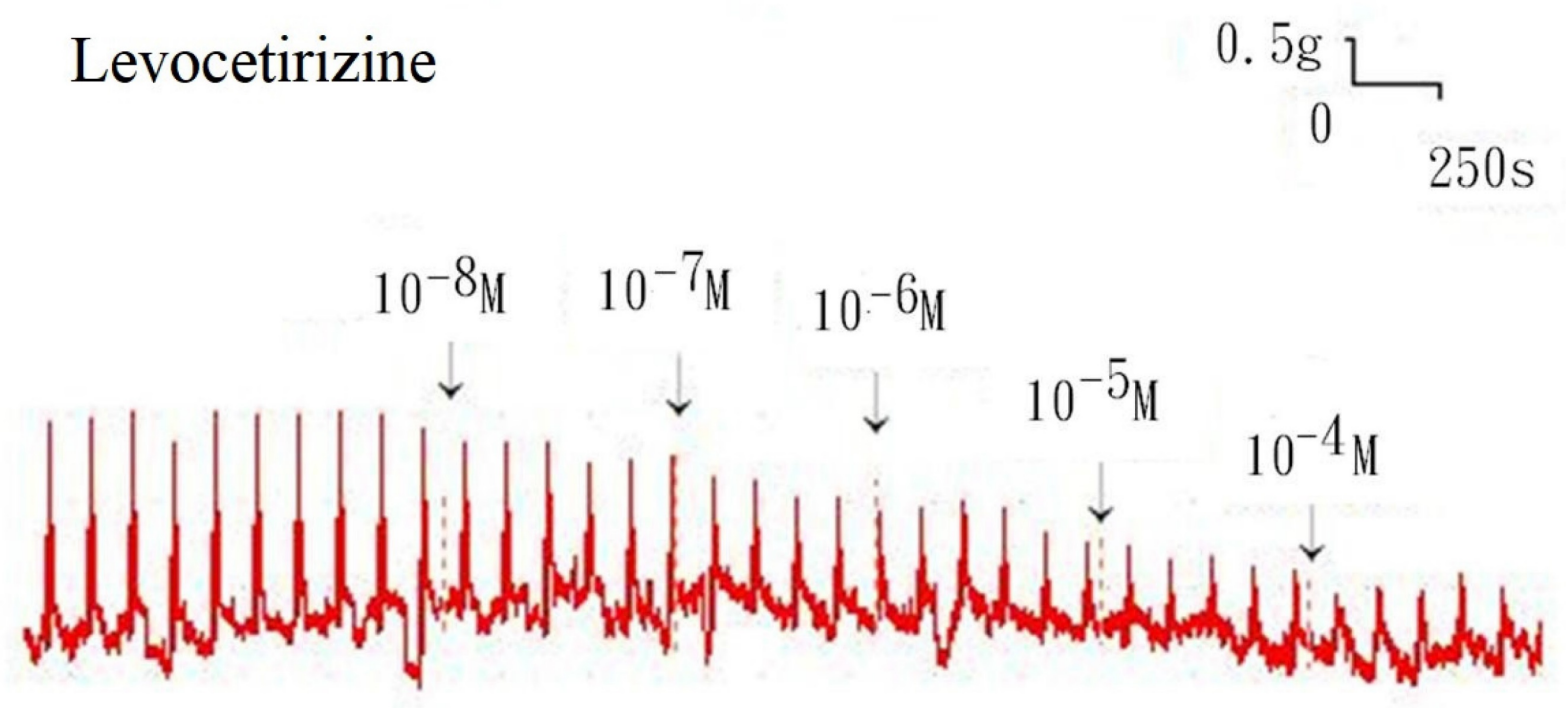
Original recording of the effects of levocetirizine on electrically induced tracheal smooth muscle contractions was noted. Levocetirizine decreased the spike contraction induced by EFS.

**Figure 5:**
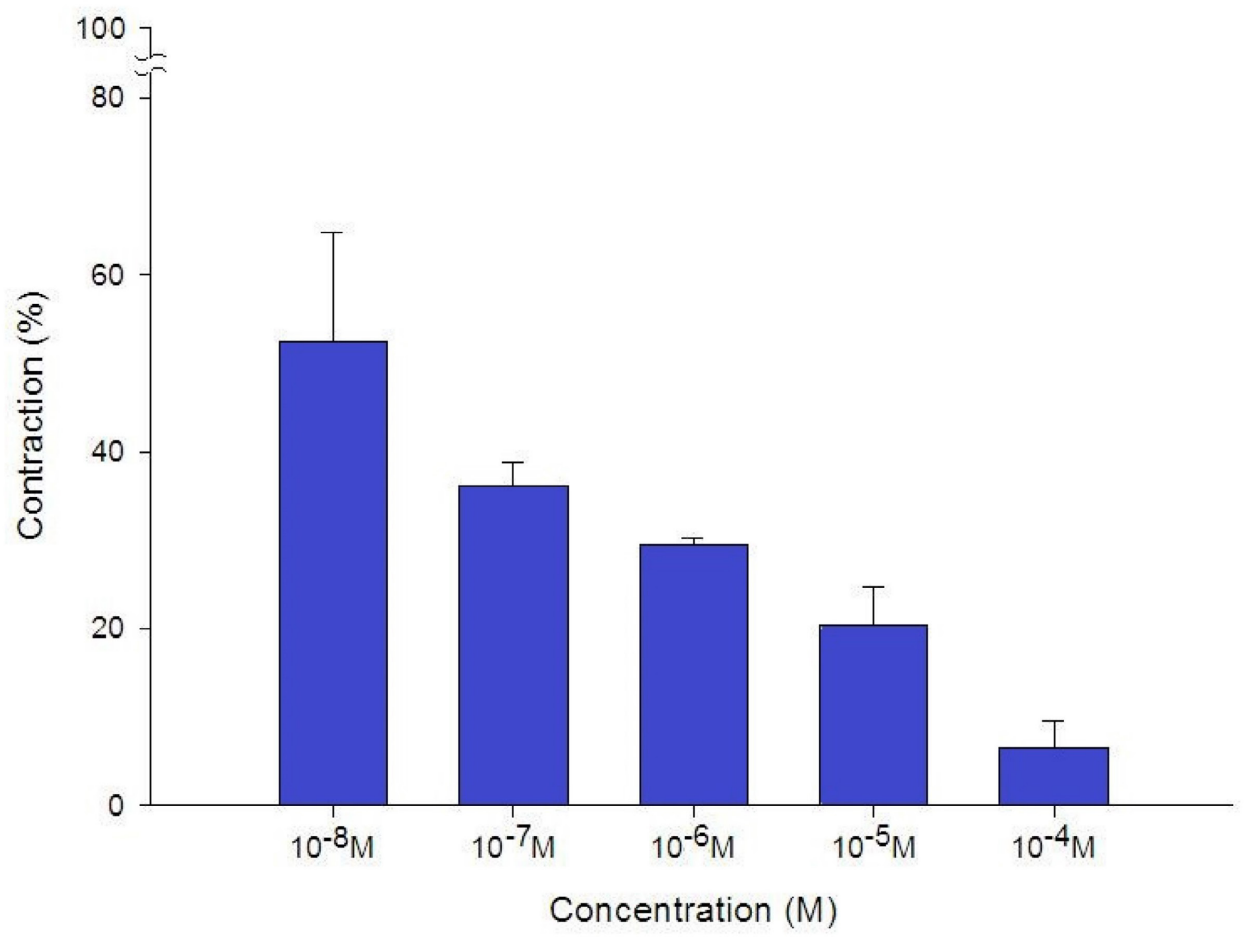
Effects of levocetirizine on electrically induced tracheal smooth muscle contractions (contraction area was calculated at 100% with no addition of levocetirizine). The peak tension values of the tracheal strip evoked by EFS during the addition of 10^-5^ M and 10^-4^ M levocetirizine were significantly lower than that with the addition of 10^-8^ M levocetirizine (P<0.001). Results were mean + SD (n = 6).

**Figure 6:**
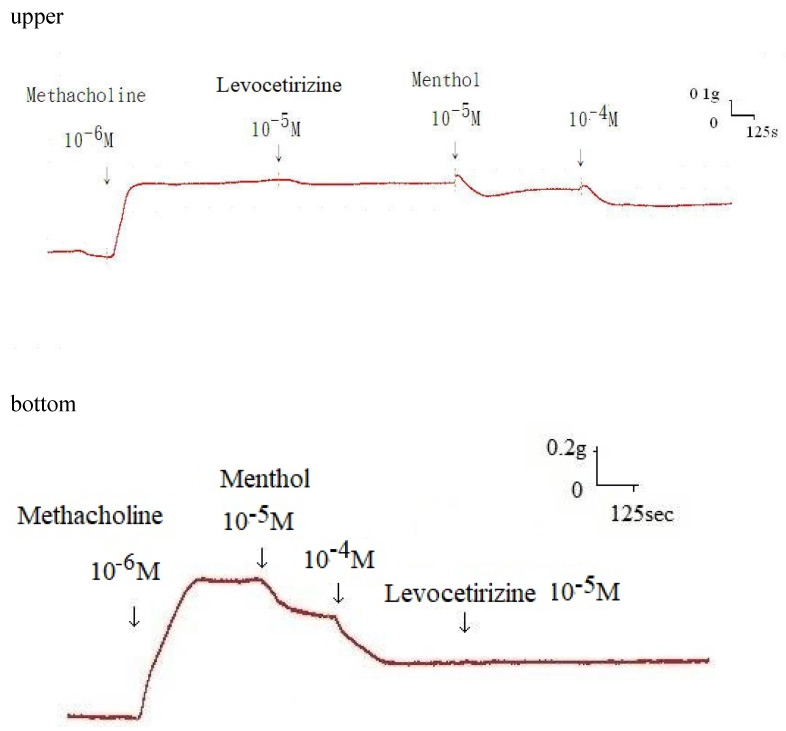
Original recording of the effects of 10^-5^ M levocetirizine on the impact of menthol either before (upper) or after (bottom) on contractions brought on by 10^-6^ M methacholine.

**Figure 7:**
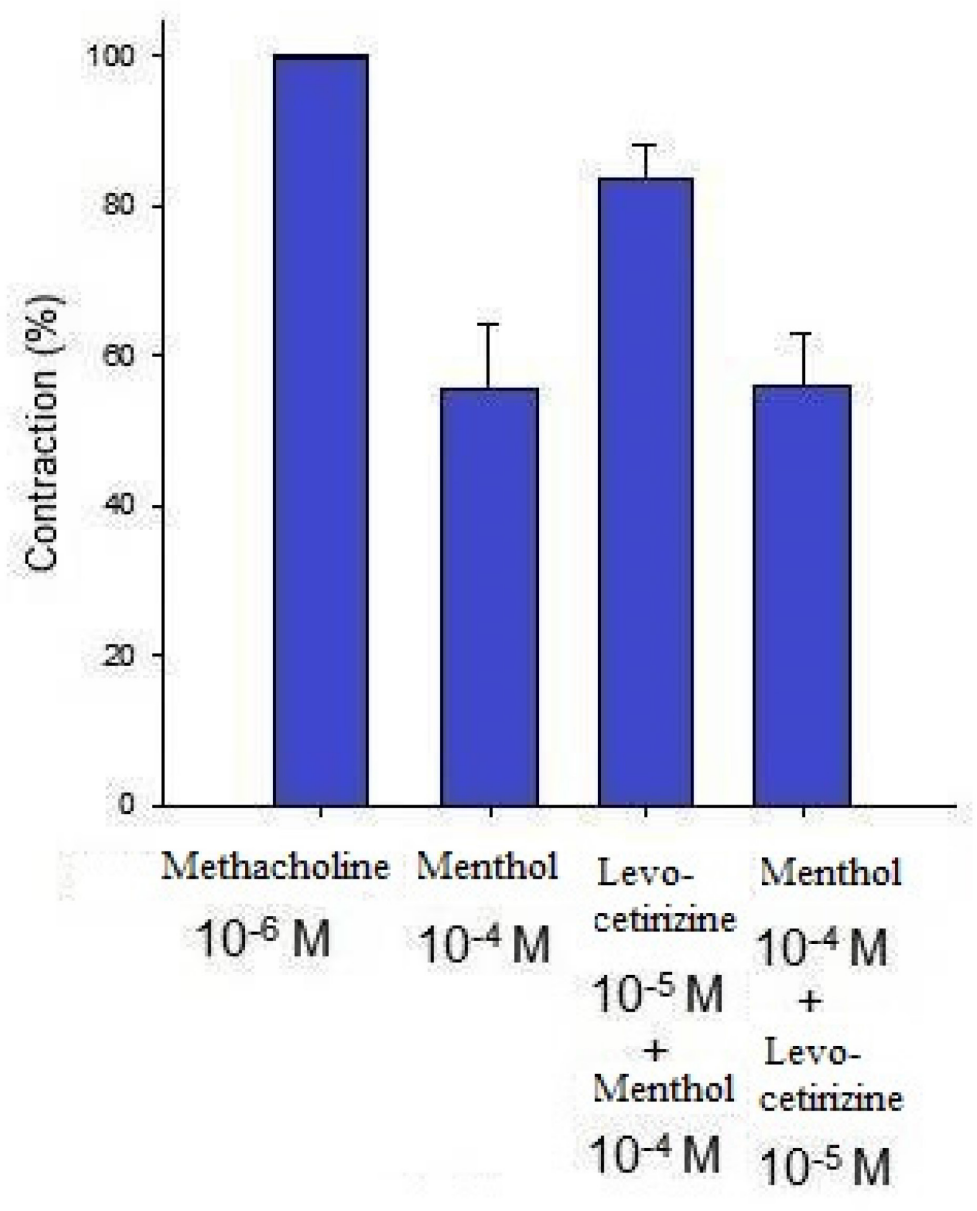
Effects of 10^-5^ M levocetirizine on the impact of menthol (either before or after) on contractions brought on by 10^-6^ M methacholine. The tensions were 83.7+4.5% and 56.2+6.8% of control values, respectively, before and after addition of 10^-5^ M levocetirizine. There was a statistically significant difference in effect of levocetirizine on menthol between before and after addition. Results were mean + SD (n = 6).

Additionally, levocetirizine prevented the spike contraction brought on by EFS (Figs. 4, 5). When 10^-8^ M levocetirizine was added, the peak tension of the tracheal strip elicited by EFS was 52.6±12.3%, while it was only 20.4±4.3% and 6.6±3.0% at 10^-5^ M and 10^-4^ M levocetirizine, respectively (Fig. 5). At 10^-5^ M and 10^-4^ M levocetirizine addition, the peak tension values of the tracheal strip elicited by EFS were considerably lower than those at 10^-8^ M levocetirizine (P < 0.001). Premedicating with 10^-5^ M levocetirizine might have lessened the effect of 10^-4^ M menthol's inhibition on contractions brought on by 10^-6^ M methacholine on the smooth muscle of the trachea, but it had no effect after 10^-4^ M menthol had already begun to operate on the tracheal strip. The tensions were 83.7±4.5% and 56.2±6.8% of control values, respectively, before and after addition 10^-5^ M levocetirizine (Fig. 6, 7). Effects of 10^-5^ M levocetirizine on the impact of menthol (either before or after) on contractions brought on by 10^-6^ M methacholine were statistically significant.

## Discussion

Only a few millimeters of trachea were needed for our test, and this trachea was removed as an intact ring. Our method relies heavily on an unbroken tracheal ring 9. Intact tracheal rings serve as significantly more accurate physiological models.

Our test required just a few millimeters of trachea, which was taken out in one piece. Our approach mainly relies on an unbroken tracheal ring 9. As physiological models, intact tracheal rings are far more precise.

However, it is important to consider the test materials employed when interpreting the findings of our investigations. The characteristics of tissues and their reactions to medicines gave some clues as to which tissue component of the trachea was responsible for drug-induced contraction, even if this was difficult to determine. First off, the tracheal strips used in our investigation were rudimentary preparations made of tracheal smooth muscle and cartilage. The other tissues (epithelium, glands, connective tissue, nerves, and cartilage) did not contract to a substantial degree, suggesting that the trachea's smooth muscle was the primary tissue component involved in contraction. Changes in tension were brought on by radial contraction of the tracheal ring since this technique entailed cross contraction. The contractile response shown in this investigation was likely an amalgam of the reactions of numerous types of muscle tissue, even though responses to medicines and electrical stimulation had been established for comparable preparations 12-14. Second, the isolated tracheal preparations that were removed from the rats for our tests did not harm the endothelium or the smooth muscle. It was safe to presume that the tracheal responses to the test chemicals in our investigation were similar to those seen after spraying the trachea during an asthma episode.

The idea of a united airway, in which allergic rhinitis and asthma can be seen as the same disease affecting various regions of the airways, has been widely supported by studies over the past ten years. After an immunologic challenge, histamine is released, which is an important factor in the inflammatory response that leads to smooth muscle contraction, vasodilation, mucus hypersecretion, and edema. H_1_-antihistamines may have concentration-dependent bronchodilator and anti-inflammatory actions. In a double-blind, placebo-controlled study, cetirizine 20 mg significantly decreased the severity of allergic rhinitis, mild to moderate perennial asthma, and nocturnal asthma symptoms in patients with allergic rhinitis over a 26-week period 15. According to a previous analysis, patients with comorbid allergic rhinitis and asthma should utilize second-generation H_1_-antihistamines since they have the best therapeutic ratio and the lowest risk of side effects at high doses 6. Our findings also showed that levocetirizine could lessen the contraction of the tracheal smooth muscle caused by methacholine. It should be emphasized, however, that the antagonized effect might also help to lessen symptoms of asthma.

The cholinergic contracting agent utilized in this formulation has a long history of use in scientific studies. It is notable that the tissue relaxation caused by levocetirizine requires a previous methacholine-induced partial contraction of smooth muscle. As a result, we should be able to evaluate the effects of popular medications and potential therapeutic agents that are said to be effective in treating asthma. Levocetirizine has several pharmacokinetic characteristics that make it an ideal antihistamine since they combine efficacy and safety. Its quick and broad absorption, constrained distribution, and extremely low degree of metabolism are what give it its clinical benefits 16. The plasma Cmax (maximal concentration) after oral administration of a single dose of levocetirizine, 5-10 mg, is around 270-559 ng/ml 17. Which translates to roughly 10^-6^ M. According to this study, levocetirizine concentrations more than 10^-5^ M might modestly reduce contractions induced on by methacholine. It is challenging to obtain the 10^-5^ M levocetirizine in accordance with its pharmacokinetic characteristics in the in vivo condition. Unless the patient takes more than 100 mg of levocetirizine. The clinical relevance is still in question. Furthermore, compared to the in vitro setting, the in vivo condition is far more complex. Although it was not obvious how levocetirizine affected the trachea smooth muscle, it was considered to as a second generation H_1_ antagonist. To fully understand this matter, more study is necessary.

The nerve terminals within the tissue being examined are activated by electrical field stimulation, which also lead to the release of endogenous neurotransmitters, which causes the smooth muscle to contract. After ipsilateral cervical sympathetic ganglionectomy, the EFS-induced spike contraction of the canine nasal mucosa that is thought to be caused by the contraction of vascular smooth muscles vanished 18. As a result, it was demonstrated that sympathetic innervation mediates EFS-induced spike contraction of isolated canine nasal mucosa 18. This study hypothesized that the activation of parasympathetic innervation caused the EFS-induced spike contraction of the tracheal smooth muscle. As a result, as the levocetirizine concentration rose, the tracheal contraction brought on by EFS decreased. These results imply that an H_1_ antagonist might inhibit the parasympathetic innervation that causes the contraction of the smooth muscles of the trachea. Clearly, the findings of this study are quite intriguing, but further research is required to fully understand these phenomena.

Histamine produced from mast cells may reach tissue concentrations of 10^-5^ to 10^-3^ M during an acute hypersensitive reaction. Although peak concentrations of an H_1_ antagonist rarely reach 10^-6^ M, they can vary depending on their physicochemical characteristics, pharmacokinetic action, and dose 19, 20. Exercise, cold or dry air hyperventilation, hypertonic or hypotonic saline, distilled water, or allergens may all cause bronchospasm, which may be at least partially prevented by pretreatment with an H_1_ antagonist. The H_1_ antagonist employed, the dose, and the stimulus used all affect how much protection is provided against these assaults 21-23. Similar to other antihistamines 24, levocetirizine might be used to treat asthma, particularly at high doses. Glucocorticoids alone had no anticholinergic effect, but that they could quickly relax the smooth muscle of the trachea when combined with antihistamines. In patients with allergic rhinitis with an acute asthma attack, using inhalation glucocorticoids and antihistamines simultaneously may be an option 25.

H_1_ antagonists reduce the mild symptoms of persistent asthma, but the impact has little therapeutic significance. Only doses greater than those for treating allergic rhinitis typically have this therapeutic effect. This investigation disproved earlier worries about possible bronchoconstriction, drying of secretions, or other harmful effects of H_1_ antagonists in asthma, which are still represented in warnings mandated by the Food and Drug Administration of the United States of America.

Today, menthol is a component of food, drink, tobacco, and cosmetic products. It is acknowledged as a naturally occurring cold receptor agonist that opens the skin's and mucous membranes' TRPM8 channels 26. In everyday life, this cold receptor agonist has been applied as a nasal inhalation solution. Menthol may impede the parasympathetic function of the trachea, according to a previous study 27. To counteract the constriction caused by 10^-6^ M methacholine, menthol at concentrations of 10^-5^ M or higher were added. This study demonstrated that 10^-5^ M levocetirizine could suppress the relaxation response to menthol-induced contraction caused by 10^-6^ M methacholine. Therefore, levocetirizine might prevent the cold receptors from working. However, if menthol acted first, the function could not be recovered. Although the findings of this study are quite intriguing, more research is required to fully understand these phenomena.

## Conclusion

Our findings showed that a high quantity of levocetirizine had an antagonized effect of methacholine-induced tracheal smooth muscle contractions. Levocetirizine might potentially impede the parasympathetic function of the trachea. Although these characteristics were not further investigated, levocetirizine might have therapeutic implications for easing asthma-related symptoms. The function of cold receptors might be inhibited by levocetirizine premedication.
